# The situation and influencing factors of outpatient satisfaction in large hospitals: Evidence from Henan province, China

**DOI:** 10.1186/s12913-021-06520-2

**Published:** 2021-05-25

**Authors:** Weicun Ren, Lei Sun, Clifford Silver Tarimo, Quanman Li, Jian Wu

**Affiliations:** 1 College of Public Health, Zhengzhou University, Zhengzhou, People’s Republic of China; 2 College of Sanquan, Xinxiang Medical University, Xinxiang, People’s Republic of China; 3 Department of Science and Laboratory Technology, Dares Salaam Institute of Technology, Dares Salaam, Tanzania

**Keywords:** Outpatient satisfaction, Large hospitals, Structural Equation Model, Dynamic Matter-Element Analysis, China

## Abstract

**Background:**

The level of outpatient satisfaction plays a significant role in improving the quality and utilization of healthcare services. Patient satisfaction gives providers insights into various aspects of services including the effectiveness of care and level of empathy. This study aimed to evaluate the level of patient satisfaction in the outpatient department and to explore its influencing factors in large hospitals (accommodating over 1000 beds) of Henan province, China.

**Methods:**

We analyzed data from Henan Large Hospitals Patient Satisfaction Survey conducted in the year 2018 and included 630 outpatients. Structural Equation Model (SEM) was used to explore the relationship among evaluation indicators of outpatient satisfaction levels. We used Dynamic Matter-Element Analysis (DMA) to evaluate the status of outpatient satisfaction. Binary Logistic Regression (BLR) was adopted to estimate the impact of personal characteristics towards outpatient satisfaction.

**Results:**

The overall score for outpatient satisfaction in large hospitals was 66.28±14.73. The mean outpatient satisfaction scores for normal-large, medium-large, and extra-large hospitals were 63.33±12.12, 70.11±16.10, 65.41±14.67, respectively, and were significantly different (*F* = 11.953, *P* < 0.001). Waiting time, doctor-patient communication, professional services, and accessibility for treatment information were shown to have directly positive correlations with outpatient satisfaction (*r* = 0.42, 0.47, 0.55, 0.46, all *P* < 0.05). Results from BLR analysis revealed that patients’ age and frequency of hospital visits were the main characteristics influencing outpatient satisfaction (*P* < 0.05).

**Conclusions:**

The outpatient satisfaction of large hospitals is moderately low. Hospital managers could shorten the waiting time for outpatients and improve the access to treatment information to improve the satisfaction of outpatients. It is also necessary to enhance service provision for outpatients under the age of 18 as well as the first-time patients.

## Background

Outpatient satisfaction is closely related to the quality of medical services provided [1–3]. However, the level of satisfaction may not only be a direct reflection of the quality and efficiency of medical service supplied, but also it may indirectly affect the government strategies in providing such services. Due to the continuous growth of global population in major developed countries, the health resources continue to be limited. The level of satisfaction with regard to healthcare services has attracted more and more attention to medical systems around the world [4–6].

As one populous country with more than 1.4 billion people, China is now experiencing a gradual increase in aging population which is occurring in a significant rate compared to many other countries [7]. The average annual growth rate of annual outpatient visits in Chinese hospitals was 5.73% between the years 2012 and 2018 [8]. Due to the current outpatient diagnosis and treatment policy, patients in China often do not need to be approved or recommended by doctors in lower-level hospitals to attend the outpatient clinics of large hospitals (with over 1000 beds) [9].

In China, the government implements a three-grade and ten-level management system for hospitals basing on the bed capacity of hospital. The grade category of the hospital determines the scope of hospital services, government financial support, and attractiveness to talents. Under normal circumstances, the bed capacity of hospital is directly proportional to its service quality, patient trust, and income [10]. Large hospitals have an important position in the overall outpatient service due to their high reputation and available technical expertise. As one of the typical provinces in central China, Henan Province has 81 large hospitals serving a population of more than 100 million people. The number of annual outpatient visits in Henan hospitals increased from 131.08 million to 207.14 million between the years 2012 and 2018. The average annual growth rate was reported to be 6.45% [11].

Existing researches suggest that outpatient satisfaction with healthcare could be explained through both providers’ and the patients’ perspective [1]. At the providers’ level, factors such as physicians’ clinical experience and hospitals’ environment might affect the outpatients’ perception towards healthcare services provided [12]. Similarly, outpatient-level factors such as demographic characteristics, motivation, and economic status play an important role in hospital services evaluation [13, 14]. One recent study found that the education status, occupation and annual income were significantly associated with overall patient satisfaction [14, 15]. Satisfaction with medical services also includes cognitive and emotional aspects, which is related to patients’ previous medical experiences [16–18].

Previous studies have reported that the quality of care received by outpatients may be a strong indicator of outpatient experiences [19, 20]. Among other important factors, hospital scale, patient-provider communication and accessibility of information have been found to be associated with outpatient satisfaction levels [21, 22]. Many Chinese hospitals have been focusing on providing patient-centered care but the evidence for its quality is still inconclusive [23, 24]. Wang and colleagues examined the relationship between patient experience and patient-centered care in public hospitals in China [19, 25]. Although it was extensive, the study did not examine important organizational characteristics on outpatient satisfaction including hospital scale. Prior evidence suggests that hospital scale is an important determinant of quality of care as well as outpatient experience [22, 26, 27].

In this regard, the current study aimed to construct a model that evaluates outpatient satisfaction and assess the relationship between the scale of hospital and outpatients’ level of satisfaction considering both patient and hospital characteristics. The results are likely to help policy makers in identifying the least satisfied group among outpatients, finding out the factors affecting satisfaction and hence providing a basis for further improvement in the quality of outpatient services in large hospitals.

## Methods

### Data sources

Patients’ information was retrieved from a cross-sectional investigation on service status and patient experience, Henan Large Hospitals Patient Satisfaction Survey in 2018 (HLHPSS 2018), which used a multistage sampling design. There are 81 large hospitals (with over 1000 beds) in Henan Province, and they are all classified as third-grade hospitals according to the three-grade and ten-level management system for hospitals in China. Of these, 13 large hospitals (located in four cities: Zhengzhou, Luoyang, Kaifeng, and Xinxiang) were selected by stratified random sampling. The survey was conducted by a team of graduate students majoring in public health, and all interviewers were trained by a specialist before the formal survey. Outpatients who agreed to participate and were able to answer all questions on their own were randomly selected for investigation in the outpatient department. During the survey, outpatients under the age of 18 were under the guidance of their parents or other guardians. All information was presented in a structured questionnaire and collected through face-to-face interview. The study was evaluated and approved by the Bioethics Committee of Zhengzhou University, and the research contents and procedures of the project complied with the international and national ethical requirements for biomedical research Cross-audit and double-entry were completed on the same day to ensure data quality. Sample size was determined by using a formula N = Z^2^×(p×(1-p))/E^2^ [28]. Among them: N is the sample size; Z is the level of confidence; E is the power of hypothesis; p is the probability value, generally of 0.5, with a confidence level of 95% and a power of hypothesis of 85% [29]. Expand the hypothetical sample size by 30% to carry out the survey, and 700 inpatients and 700 outpatients were included in HLHPSS 2018. 36 outpatients failed to complete the survey due to queuing, to be examined and other related matters, while 10 questionnaires were filled with potential errors, and 24 questionnaires were excluded due to missing responses. In the end, 630 (N) outpatients were included in this study. Based on the average daily number of outpatient visits in 13 hospitals in 2017, 5.46% of outpatients participated in the survey, and 4.96% of outpatients were included in the final analysis.

Hospital information, including average annual open beds, number of outpatient visits and number of employees in the department were obtained from the 2017 Annual Statistic Book of Henan Health and Family Planning Commission. According to the bed capacity (n), the 13 large hospitals were divided into three scale categories: Normal-large hospitals (NH) (1000 < n ≤ 1500), Medium-large hospitals (MH) (1500 < n ≤ 3000), and Extra-large hospitals (EH) (n > 3000) (Table 1).

**Table 1:** Characteristics of the 13 large hospitals in Henan, China (2017)

Hospitals	Average annual open beds (beds)	Hospital outpatient visits (10000 persons)	Number of discharged (10000 persons)	Number of employees (persons)	Number of doctors (persons)	Medical income (billion yuan)
**NH**^c^						
H1	1024	11.36	1.13	1031	262	2.38
H2	1092	37.07	2.42	1541	477	3.77
H3	1104	10.78	1.99	1281	353	6.69
H4	1211	13.92	2.81	1460	437	4.04
**MH**^d^						
H5^a^	1687	37.34	7.29	1152	538	15.11
H6	1778	49.05	4.82	2006	671	12.05
H7	1922	37.93	4.81	1929	601	10.91
H8	2047	39.91	6.30	2218	610	10.31
H9	2978	42.67	9.31	3245	951	15.35
**EH**^e^						
H10	7659	73.59	52.82	7992	3018	134.72
H11	4437	51.04	22.86	7007	1974	58.49
H12	3564	44.45	11.41	3509	1064	38.22
H13^b^	3212	19.98	14.36	3112	774	30.82

^a^H5 is a specialized hospital for women and children; ^b^H13 is an oncology hospital; ^c^NH: Normal-large hospitals; ^d^*MH* medium-large hospitals; ^e^*EH* extra-large hospitals.

### Evaluation model for outpatient satisfaction

The survey draws on the “Patient Satisfaction Questionnaire III Edition” (PSQIII) from RAND [30] and formed an item pool containing 24 items based on China’s actual situation through expert consultation and brainstorming. The survey covered seven dimensions of outpatient satisfaction including: basic information of outpatients, hospital environment and facilities, waiting time, patient-provider communication, professional services, accessibility of treatment information and overall satisfaction. For each question, there are five standard options, namely: “very satisfied”, “satisfied”, “average”, “dissatisfied”, and “very dissatisfied”. In addition to the basic information of outpatients, these options were assigned scores from 1 to 5 following the Likert Five-point Scale. The preliminary designed questionnaire was tested by a pre-survey involving 70 outpatients in large hospitals, and all respondents completed the questionnaire within 10 minutes. According to the pre-survey results, two less effective items including hospital reputation and medical expenses have been deleted, and the degree of patience in questionnaire has been revised to be easily understood for outpatients [31]. Finally, the questionnaire for outpatient satisfaction of large hospitals contained 7 dimensions and 22 items. The Cronbach’s α coefficient for questionnaire reliability and content validity index (CVI) were 0.761 and 0.798, respectively.

#### Outcome variables

Participants in the survey were asked about their feelings during the whole medical treatment they received from the specified hospital. The main outcome variable overall satisfaction (Y6) was measured by using three key indicators which were (1) level of understanding of personal illness (x16): level of understanding of personal illness after seeing a doctor; (2) evaluation of overall treatment process (x17): which refers to the overall evaluation of self-perception during the treatment; (3) personal feelings on time spent during the visit (x18).

#### Independent variables

The independent variables were divided into five categories: (1) Environment and facilities (Y1) which was derived from cleanness and tidy condition (x1), availability of necessary facilities (x2) and signage settings (x3); (2) Waiting time (Y2) obtained from number of medical appointments (x4), waiting time for a doctor (x5) and waiting time for medical results (x6); (3) Doctor-patient communication (Y3) derived from degree of patience (x7), level of details communicated (x8) and the length of communication (x9); (4) Professional services (Y4) including medical consultation (x10), sense of responsibility (x11) and professional level attained (x12); (5) Accessibility of treatment information (Y5) obtained from drug information (x13), precautions (x14) and medical expenses (x15).

#### Structural Equation Model (SEM)

**Fig. 1:**
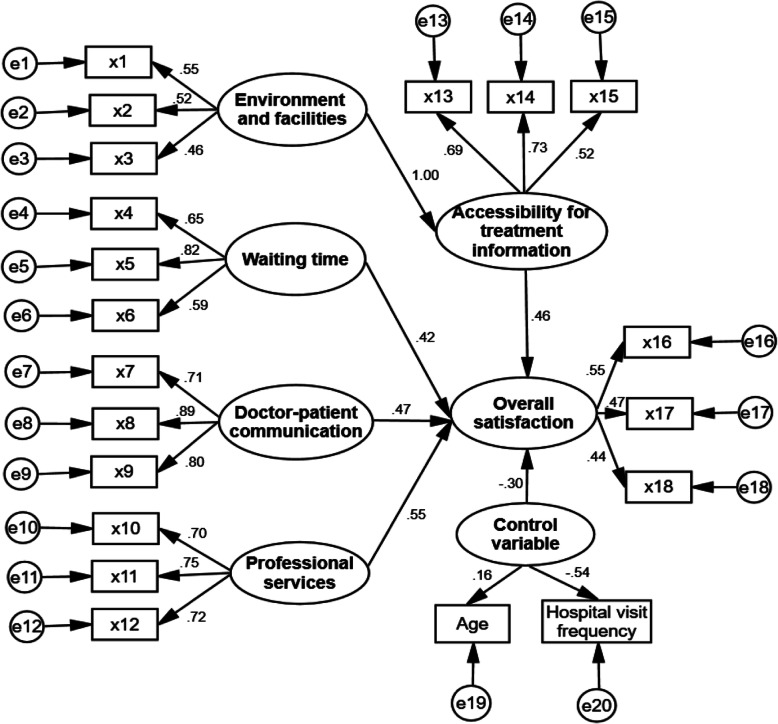
Evaluation model of outpatient satisfaction in 13 large hospitals in Henan

Structural Equation Model (SEM) was used to analyze the relationship among the evaluation indicators of outpatient satisfaction. A SEM model which includes all the relationships between indicators was constructed based on the on-site survey data of outpatient satisfaction. Age, sex, educational level, and frequency of hospital visit were also included in the model as control variables. Indicators that showed a non-significant relationship (*P* > 0.05) were removed. The corrected evaluation model of outpatient satisfaction with correlation coefficient (*r*) between indexes was shown in Fig. 1.

The fitting index of the satisfaction evaluation model was 1.93, which was less than 2 and close to 1. The sample co-variance matrix was close to the estimated co-variance matrix. At the same time, the calculation results of the model show that the root-mean-square error of approximation = 0.047 < 0.05, and the value of goodness of fit index, adjusted goodness of fit index, incremental fit index, normative fit index, comparative fit index were all greater than 0.80, indicating that the fitting effect of the model was acceptable but not very good. This situation may be caused by sample size which is considered acceptable for path analysis but not large and the nature of control variables which may have reduced the goodness of fit of the final model.

#### Dynamic Matter-element Analysis (DMA)

Dynamic Matter-element Analysis (DMA) is an objective evaluation method that compares the evaluation results with the theoretical optimal values [32]. With the reward and penalty function which reduces the influence of extreme values by assigning larger values to smaller weights, the dynamic weight can be specific to each secondary evaluation index, making the evaluation result more accurate. To apply DMA to analyze the survey data of each indicator in the model Involves the following indicators. Firstly, the reward and penalty function value (d_i_):

$$ {d}_i=\frac{1}{x_i} $$

where *x*_*i*_ is the evaluation value of each individual indicator.

Secondly, the sum of the reward and penalty function values of all individual indicators (l):

$$ l=\sum \limits_1^6{d}_i $$

Finally, the weight value of each indicator (W_i_):

$$ {W}_i=\frac{d_i}{l} $$

Each indicator represents a matter dimension, therefore we could construct a 15-dimensional matter element *R* for outpatient satisfaction evaluation. With reference to the relative optimization criterion stating “the bigger the better”, the maximum value of the survey results of each index was selected to construct a new matter element among the 15-dimensional complex matter elements of 630 outpatients, which was defined as the best outpatient satisfaction matter element *R*_*0*_. The correlation coefficient of the *i*_*th*_ evaluation index between the *j*_*th*_ outpatient and *R*_*0*_ was:

$$ {L}_{ij}=\frac{\Delta_{\mathrm{min}}+\rho {\Delta}_{\mathrm{max}}}{\Delta_{ij}+\rho {\Delta}_{\mathrm{max}}} $$

Where: *Δ*_*ij*_ is the absolute difference between the value of the *i*_*th*_ evaluation index of the best satisfaction outpatient and the corresponding index value of the *j*_*th*_ medical community (*i* = 1, 2, ..., 15; *j* = 1, 2, ..., 630). *Δ*_*min*_ is the minimum value of the absolute difference of the index value *Δ*_*ij*_. *Δ*_*max*_ is the maximum value of the absolute difference of the index value *Δ*_*ij*_; *ρ* is the resolution coefficient, which is 0.5 generally.

Comprehensive measurement of the correlation between the satisfaction of each outpatient and the best outpatient satisfaction was *L*_*0j*_ (satisfaction evaluation result):

$$ {L}_{0j}=100\sum \limits_{j=1}^{10}\sum \limits_{i=1}^6{W}_i{L}_{ij} $$

Where: *L*_*0j*_ is between 0 - 100, and the closer it is to 100, the better the evaluation result is.

### Statistical analysis

The evaluation model was constructed by using SEM. DMA was used to evaluate the satisfaction of outpatients. One-Way Analysis of Variance was used to compare the mean differences in outpatient satisfaction scores among different hospitals. And the influencing factors for outpatient satisfaction was analyzed by using Binary Logistic Regression (BLR). *P*-values of < 0.05 were considered statistically significant. Data was double entered using Epidata 3.0 software. SPSS 20.0 and Amos 22.0 software were used for statistical analysis.

## Results

### Basic information of outpatients

The descriptive characteristics of study participants are listed in Table 2. The average age of study subjects was 40 years. 58% constituted of females while 42% were males. Participants under 18 years and over 60 years accounted for 12.32% and 46.45%, respectively. There was a statistically significant difference in satisfaction among age groups (*F* = 3.236, *P* = 0.040). More than 73% of the outpatients attended senior middle school and below while over 47% visited the hospital for the first time. At the same time, the analysis found that there was no statistical difference in the distribution of age, sex, educational level and hospital visit frequency of outpatients among NH, MH and EH (*P* > 0.05).

**Table 2:** Characteristics of study participants (*N* = 630)

Index	Outpatients (person (%))	Satisfaction (mean±SD)	*t/F*	*P*
**Age (years)**				
<18	60 (12.32)	59.75±7.97	3.236	0.040
18-60	246 (41.23)	66.67±15.01		
>60	294 (46.45)	66.98±14.42		
**Sex**				
Male	264 (42.18)	65.51±13.58	-1.298	0.195
Female	366 (57.82)	67.02±15.49		
**Educational level**				
Junior high school and below	375 (59.24)	65.30±13.27	2.179	0.089
Senior middle school	90 (14.69)	68.20±16.55		
Junior college	135 (21.33)	68.51±16.72		
Bachelor degree and above	30 (4.74)	64.95±15.91		
**Hospital visit frequency (times)**				
1	285 (47.87)	63.27±14.78	9.430	<0.001
2	147 (25.12)	67.90±14.78		
3	84 (13.74)	72.70±15.04		
≥4	84 (13.27)	66.10±13.38		

### Relationship between satisfaction evaluation indexes based on hospital bed capacity

**Fig. 2:**
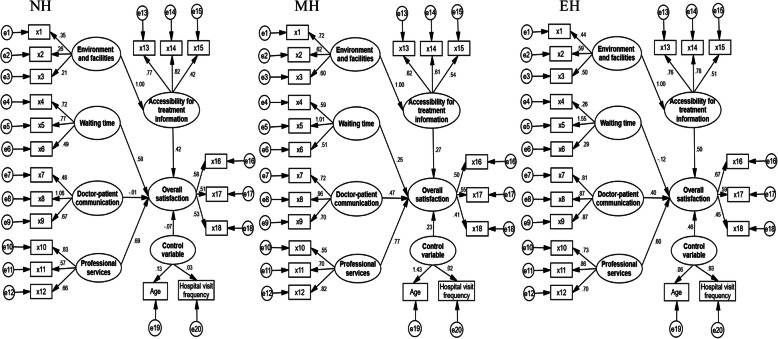
Satisfaction evaluation model of outpatients in different scale hospitals. NH: Normal-large hospitals; MH: Medium-large hospitals; EH: Extra-large hospitals

Substituting the outpatient satisfaction survey data of the three types of hospitals into the SEM constructed in the Methods section, we worked out the relationship (r) between satisfaction evaluation indicators (Fig. 2). The indicators with the strongest correlation were all Professional services (*r* = 0.69, 0.77, 0.60) in 3 types of hospitals, while the indicators with the weakest correlation were doctor-patient communication, waiting time, and waiting time (*r* = -0.01, 0.25, -0.12) in NH, MH, and EH. The correlation between control variables and the overall satisfaction were -0.07, 0.23, and 0.46, respectively.

### Objective evaluation of satisfaction based on DMA

#### Dynamic weights calculation

**Fig. 3:**
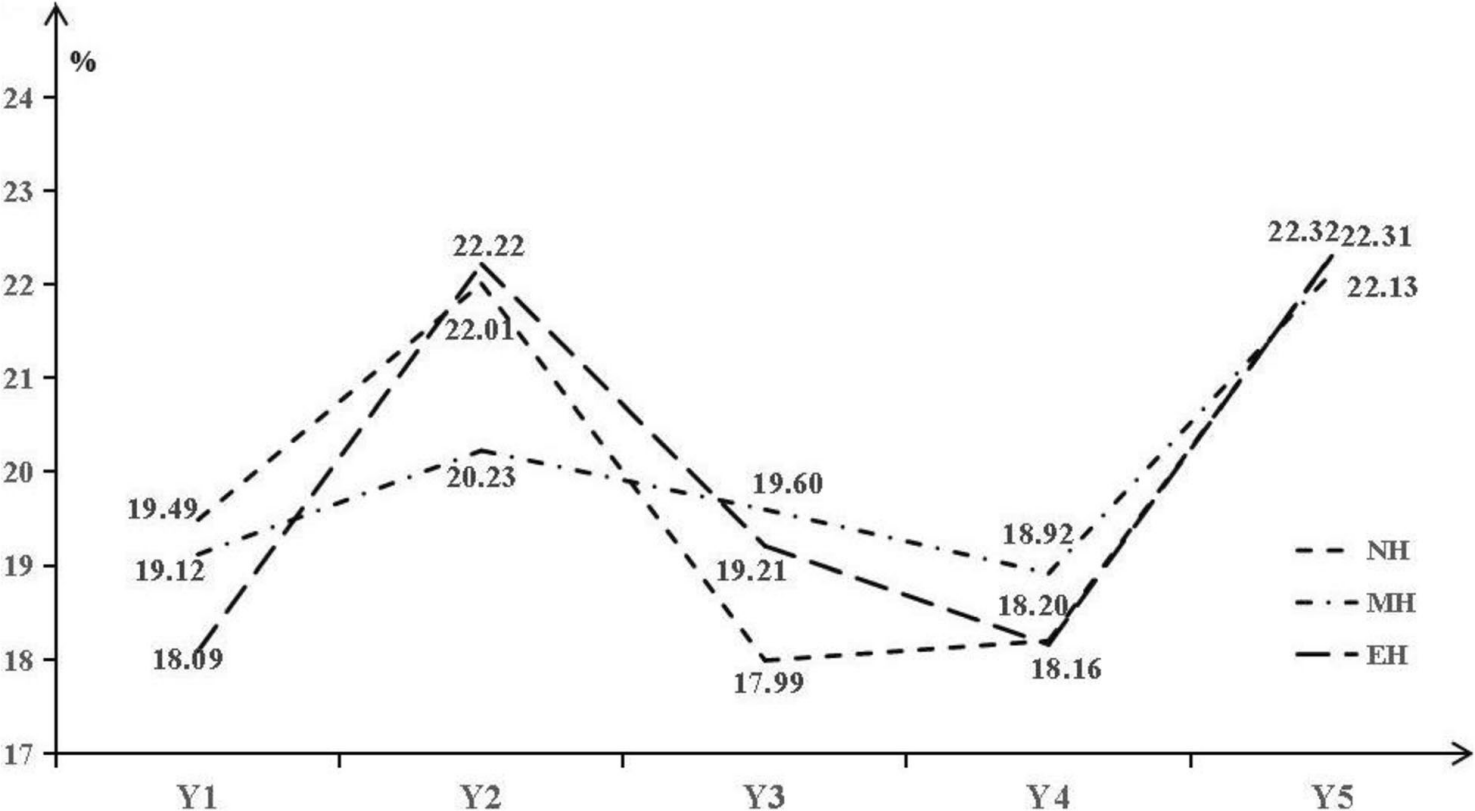
Dynamic weights of evaluation indicators. Y1: Environment and facilities; Y2: Waiting time; Y3: Outpatient-provider communication; Y4: Professional services; Y5: Accessibility for treatment information; NH: Normal-large hospitals; MH: Medium-large hospitals; EH: Extra-large hospitals

Dynamic weights of evaluation indicators were shown in Fig. 3. Among the five indicators, accessibility for treatment information had the highest weight (22.25%) while the professional services attained the lowest (18.43%). Our results showed that the weights of evaluation dimension in NH, MH and EH were not the same: In NH, accessibility for treatment information occupied the largest weight (22.31%) while the doctor-patient communication occupied the smallest (17.99%); In MH, accessibility for treatment information weighted 22.13% and professional services occupied the smallest weight (18.92%); While in EH, accessibility for treatment information and environment and facilities occupy the largest (22.32%) and smallest (18.09%) weights, respectively.

#### Outpatient satisfaction evaluation results

The evaluation results of outpatient satisfaction in large hospitals are shown in Table 3. The overall evaluation scores of outpatient satisfaction in NH, MH and EH were 63.33±12.12, 70.11±16.10, 65.41±14.67, respectively. The differences in mean scores among the three hospital categories were statistically significant (*F* = 11.953, *P* < 0.001). Among the five evaluation indicators, only doctor-patient communication dimension showed that the difference in mean scores of the three types of hospitals were not statistically significant (*P* > 0.05). MH’ outpatient satisfaction evaluation was relatively optimal in the other four dimensions (*F* = 19.337, 14.552, 12.909, 7.065, all *P* < 0.05). And the satisfaction of outpatients in EH were better than that of NH in the dimensions of environment and facilities as well as professional service.

**Table 3:** Outpatient satisfaction evaluation results

Indicators	NH^a^	MH^b^	EH^c^	*F*	*P*
Environment and facilities	12.26±2.88	14.32±3.69	13.65±3.52	19.337	<0.001
Waiting time	12.38±2.70	13.73±3.71	12.25±2.93	14.552	<0.001
Doctor-patient communication	13.40±3.17	14.02±3.88	13.33±3.68	2.384	0.093
Professional services	12.85±3.03	14.57±3.54	13.76±3.58	12.909	<0.001
Accessibility for treatment information	12.44±2.91	13.46±3.89	12.42±2.95	7.065	0.001
**Overall satisfaction**	**63.33±12.12**	**70.11±16.10**	**65.41±14.67**	**11.953**	**<0.001**

^a^*NH* normal-large hospitals, ^b^*MH* medium-large hospitals, ^c^*EH* extra-large hospitals

### Analysis of influencing factors based on BLR

A BLR method was used to analyze the influencing factors for outpatient satisfaction. According to univariate analysis and the existing research results of outpatient satisfaction [3, 16], age, sex, educational level, and the number of hospital visits were included as independent variables in the regression model to avoid false negatives. In this study, outpatient satisfaction degrees were classified into two categories, and the results of each index were assigned a value of 0 or 1 according to the score. See Table 4 for values assigned to variables.

**Table 4:** Variables assignment

Variables	Assignment
Outpatient satisfaction	< 60 points = 0, ≥ 60 points = 1
Age	< 18 = 1, 18 ≤ Age ≤ 60 = 2, >60 = 3
Sex	Male = 0, Female = 1
Educational level	Junior high school and below = 1, Senior middle school = 2, Junior college = 3, Bachelor degree and above = 4
Hospital visit frequency	First = 0, Second = 1, Third = 3, Fourth and above = 4

Regression analysis results showed that the satisfaction of outpatients in large hospitals was mainly affected by patients’ age and hospital visit frequency (*OR =* 2.395, 2.340, 1.596, 0.368, all *P* < 0.05). Specific to the three types of hospitals, the outpatients’ age did not have a significant impact on outpatient satisfaction in NH (*P* > 0.05). While in EH, outpatient satisfaction was also affected by the patient’s educational level in addition to patient age and frequency of visits (*OR* = 0.118, 0.114, 0.203, all *P* < 0.05). See Table 5.

**Table 5:** Binary Logistic Regression (BLR) results

Variables	Total			Three scale categories hospitals								
				NH^a^			MH^b^			EH^c^		
	*P*	*OR*	*95%CI*	*P*	*OR*	*95%CI*	*P*	*OR*	*95%CI*	*P*	*OR*	*95%CI*
**Age (>60 years)**												
< 18	0.048	2.395	[1.008, 5.695]	0.666	1.428	[0.284-7.183]	0.004	0.240	[0.092-0.627]	0.018	7.123	[1.395-6.366]
18 ≤ Age ≤ 60	0.900	0.969	[0.596, 1.576]	0.709	1.200	[0.460-3.131]	0.909	0.907	[0.171-4.182]	0.070	2.234	[0.935-5.337]
**Sex**	0.744	1.057	[0.757, 1.477]	0.975	1.011	[0.527-1.938]	0.611	1.188	[0.612-2.305]	0.465	1.260	[0.679-2.338]
**Educational level (Bachelor degree and above)**												
Junior high school and below	0.120	0.532	[0.240, 1.179]	0.890	1.237	[0.061-2.203]	0.717	1.304	[0.311-5.472]	0.002	0.118	[0.030-0.467]
Senior middle school	0.589	0.785	[0.326, 1.888]	0.752	1.667	[0.070-3.932]	0.223	2.578	[0.563-9.814]	0.007	0.114	[0.024-0.545]
Junior college	0.193	0.574	[0.248, 1.324]	0.493	2.940	[0.135-4.088]	0.907	0.918	[0.215-3.908]	0.038	0.203	[0.045-0.917]
**Hospital visit frequency (Fourth and above)**												
First	<0.001	2.340	[1.539, 3.558]	0.086	2.101	[0.899-4.908]	0.043	2.080	[1.024-4.227]	0.011	2.907	[1.276-6.626]
Second	0.047	1.596	[1.006, 2.533]	0.003	4.306	[1.669-9.108]	0.782	1.137	[0.457-2.833]	0.711	1.180	[0.492-2.830]
Third	0.003	0.368	[0.192, 0.707]	0.027	0.189	[0.043-0.830]	0.049	0.249	[0.063-0.994]	0.327	0.569	[0.185-1.755]
**Constant**	0.874	-	-	0.568	-	-	0.920	-	-	0.642	-	-

^a^*NH* normal-large hospitals, ^b^*MH* medium-large hospitals, ^c^*EH* extra-large hospitals.

## Discussion

The overall satisfaction of outpatients was shown to be low in large hospitals of Henan province while significant difference in level of satisfaction was observed among outpatients from the different category hospitals. In detail, outpatient satisfaction of MH was relatively the highest, and the outpatient satisfaction of EH was slightly higher than that of NH. The results also showed that the scores of doctor-patient communications were the highest among the five evaluation dimensions, and there was no statistically significant difference of scores among hospitals categories. In addition, we proved that there were significant differences in the satisfaction of outpatients about environment and facilities, waiting time, professional services, and accessibility for treatment information among the three types hospitals. However, only in the two dimensions of “environment and facilities” and “professional services”, we found that the satisfaction of outpatients in EH had a significant advantage over MH [21].

Our results showed that the initial expansion of the hospital scale had a positive impact on the satisfaction of outpatients, and the satisfaction would decrease when the scale of the hospital was expanded from MH to EH. The situation was similar in the United States whereby as the scale of the hospital expanded, outpatient satisfaction increased to a certain level. Since then, as the scale of the hospital continued to increase, the level of satisfaction would remain unchanged, and the satisfaction would eventually decline when the hospital was larger enough [22]. One likely reason for better satisfaction in MH is that, hospitals with 1500 - 3000 beds could be large enough to provide a full range of professional services and outpatient information departments, but EH is relatively so large that outpatients may sometimes get lost prior to searching for a specific service.

The results of the SEM analysis revealed that professional service was considered as the most relevant factor for outpatient satisfaction. Accessibility for treatment information and waiting time had relatively low correlations with satisfaction, which showed consistency with many other studies [11, 33, 34]. Doctor-patient communication also had a direct impact on outpatient satisfaction. Although environment and facilities were indispensable for evaluating the satisfaction of outpatients, we found that it indirectly affects satisfaction level through accessibility for treatment information.

At the same time, consistent with the analysis results of the SEM, the DMA results based on the extension set theory showed that accessibility for treatment information and professional services occupied the maximum and minimum weights, respectively. For NH, MH and EH, the evaluation dimensions with the smallest weight were doctor-patient communication, professional services and environment and facilities. In smaller hospitals, outpatients had more time and opportunities to communicate with doctors, and hence the doctor-patient communication becomes enhanced [26]. As hospital scale increases from NH to MH, outpatients’ perception of the quality of professional services in the hospital increased significantly. However, if the scale of the hospital would be expanded from MH to EH, outpatients will pay more attention to facilities and environment.

In addition, we found that age had an important influence on the satisfaction of outpatients. The relationship between age of participants and the level of satisfaction was shown to be increasing at first but eventually it was decreasing with the increase of hospital beds. Except NH, satisfaction among participants aged under 18 years was significantly lower compared to those aged over 18 years. This finding is consistent with the one conducted in China which elucidated the positive correlation between age and the level of satisfaction [19]. Since age is an unmodified factor, policymakers and organizations may choose to concentrate on enforcement of healthcare policies that supports all age categories [35]. The total influences of sex and education on satisfaction were not significant, which was inconsistent with studies elsewhere where education level of outpatients were found to affect outpatient satisfaction [14]. This may be related to the shorter outpatient visit time, shorter communication time with doctors, and higher overall quality of service in large hospitals in our study.

Finally, for outpatients, there was a significant difference in mean scores in satisfaction of outpatients with different visits to the same hospital, a scenario which may also deserves a close attention of hospital decision makers. The overall satisfaction of outpatients increased with the number of visits, but when the frequency was more than three times, the outpatients’ satisfaction was shown to decrease significantly. At the same time, the impact of the frequency of visits on satisfaction level was positively related to the scale of the hospital. The outpatients’ familiarity with the medical environment may improve their medical experience, but after being more familiar with hospital services and environment in large hospitals, patients tend to demand more additional services and hence are more likely to report unsatisfied experience [18]. Therefore, we think that patients should be given a uniform treatment when it comes to medical services regardless of their frequency of visits to that hospital. In addition, managements of specific hospitals are called upon exploring various ways to satisfy patients’ specific needs.

This study has several strengths: Firstly, we focused our research on large hospitals and outpatient satisfaction. Secondly, the model was designed with multiple outcome variables based on SEM and hence provided better explanation on the relationship between each evaluation variable and the level of satisfaction among outpatients. Thirdly, the weight distribution and evaluation results of DMA may provide evidence-based references for decision makers practicing in large hospitals to reform outpatient services department. However, a significant limitation of the study is that, the findings based on the samples selected from Henan province may not be fully applicable or generalized to other areas given the sample coverage was limited and the differences in economic levels and social characteristics. In addition, since the current study was a cross-sectional survey, we think that there may be other important confounders such as seasonal, weather and others that may play significant roles in outpatient satisfaction ratings warranting for further investigation and analysis in this specific area.

## Conclusions

Our study suggests that the overall outpatient satisfaction rating in large hospitals was moderately low and was directly affected by waiting time, doctor-patient communication, professional services and accessibility for treatment information. Findings form DMA showed that the expansion of the hospital scale helped to improve outpatient satisfaction at the initial stage, but the continuous and excessive expansion of the hospital scale had a negative impact on outpatient satisfaction. At the same time, age and number of visits are important to explain the differences in satisfaction scores among outpatients. Therefore, decision makers should take effective and targeted measures to resolve the contradiction between various factors in outpatient services, and control the excessive expansion of hospital scale, rather than just increasing resources to resolve service inadequacy.

## Data Availability

The data that supports the findings of this study is available from Health Commission of Henan Province, but restrictions are applied to the availability of the data, with license for the current study, and so it is not publicly available.

## Abbreviations

SEM: Structural equation model
DMA: Dynamic matter-element analysis
BLR: Binary logistic regression
NH: Normal-large hospitals (1000 < beds ≤ 1500)
MH: Medium-large hospitals (1500 < beds ≤ 3000)
EH: Extra-large hospitals (beds > 3000)
HLHPSS 2018: Henan Large Hospitals Patient Satisfaction Survey in 2018
